# A set of microsatellite markers with long core repeat optimized for grape (*Vitis *spp.) genotyping

**DOI:** 10.1186/1471-2229-8-127

**Published:** 2008-12-16

**Authors:** Guido Cipriani, Maria Teresa Marrazzo, Gabriele Di Gaspero, Antonella Pfeiffer, Michele Morgante, Raffaele Testolin

**Affiliations:** 1 Dipartimento di Scienze Agrarie e Ambientali, University of Udine, Via delle Scienze 208, 33100 Udine, Italy; 2 Istituto di Genomica Applicata, Parco Scientifico e Tecnologico 'Luigi Danieli' Udine, Italy

## Abstract

**Background:**

Individual fingerprinting based on molecular markers has become a popular tool for studies of population genetics and analysis of genetic diversity in germplasm collections, including the solution of synonymy/homonymy and analysis of paternity and kinship.

Genetic profiling of individuals is nowadays based on SSR (Simple Sequence Repeat) markers, which have a number of positive features that make them superior to any other molecular marker developed so far. In humans, SSRs with core repeats three to five nucleotides long are preferred because neighbour alleles are more easily separated and distinguished from each other; while in plants, SSRs with shorter repeats, namely two-nucleotides long, are still in use although they suffer lower separation of neighbour alleles and uncomfortable stuttering.

**Results:**

New microsatellite markers, containing tri-, tetra-, and penta-nucleotide repeats, were selected from a total of 26,962 perfect microsatellites in the genome sequence of nearly homozogous grapevine PN40024, assembled from reads covering 8.4 X genome equivalents.

Long nucleotide repeats were selected for fingerprinting, as previously done in many species including humans. The new grape SSR markers were tested for their reproducibility and information content in a panel of 48 grape cultivars. Allelic segregation was tested in progenies derived from two controlled crosses.

**Conclusion:**

A list of 38 markers with excellent quality of peaks, high power of discrimination, and uniform genome distribution (1–3 markers/chromosome), is proposed for grape genotyping. The reasons for exclusion are given for those that were discarded. The construction of marker-specific allelic ladders is also described, and their use is recommended to harmonise allelic calls and make the data obtained with different equipment and by different laboratories fully comparable.

## Background

Individual fingerprinting based on molecular markers has become a popular tool for studies of population genetics and analysis of genetic diversity in germplasm collections, including the solution of synonymy/homonymy and analysis of paternity and kinship.

Genetic profiling of individuals is nowadays based on SSR (Simple Sequence Repeat) markers, which have a number of positive features that make them superior to any other type of molecular marker developed so far for DNA fingerprinting [1].

SSR markers, also known as short tandem repeats (STRs) or microsatellites, consist of tandemly repeated DNA sequences with a core unit of 1–6 base pairs (bp). Beside their abundance in plant genomes, a feature that they share with other types of markers is their high level of variability in the number of repeats of the core motif, occasionally showing dozens of alleles at each locus. They are amplified by PCR using a primer pair that anneals to the repeat flanking regions and therefore tag a single locus in diploid genomes. Finally they are highly reproducible among laboratories without requiring any DNA exchange.

In humans and animals, for which fingerprinting protocols are well-established, long nucleotide repeats, namely tetra- and penta-nucleotides, are adopted [2-6] (see also ). In tetra and penta-nucleotide SSRs, neighbour alleles are more easily separated and identified from each other, while di-nucleotides are neglected mainly because of the lower separation of neighbour alleles and the high amount of stuttering, which make the interpretation of electropherograms and the call of true alleles less reliable [7,8].

Microsatellites with long core motifs are less frequent and shorter than mono- and di-nucleotides [9] and their isolation from SSR-enriched genomic libraries yielded only a few numbers of clones carrying such types of repeats [10,11], until very recently [12]. Moreover, libraries were constructed in most cases with the aim of producing markers useful for genetic mapping, and di-nucleotide markers, being by far more frequent in the genomes and easy to isolate, were better suited for this scope.

Plant scientists have therefore developed genotyping techniques based mainly on di-nucleotide repeats. These markers require very accurate and reliable protocols for allele separation and identification, to avoid allele miscalling. Weeks et al [13] reported that 83% of discrepancies between laboratories in scoring di-nucleotide alleles are due to arbitrary decisions in binning, the process that converts raw allele lengths into allele classes, and the size is then expressed by an integer.

Many different procedures of electrophoresis are used to separate SSR alleles. The method currently accepted for human DNA in forensic disputes is based on PCR carried out with dye-labelled primers; fragments are then analysed by capillary electrophoresis in automatic sequencers and alleles sized with reference allelic ladders constructed for each locus [14]. Similar protocols are being developed for animals, such as domestic dogs [5]. In plants, such a robust and reliable procedure is still rare. More simple and less expensive protocols are often adopted, in the worst cases based on the use of manually cast gels, the detection of DNA fragments by silver staining, and the estimate of allele size by comparison with anonymous ladders or plasmid sequences loaded on the gel in the adjacent lanes. The grapevine community has already taken the first step towards the adoption of allelic ladders for a limited number of markers [15].

In the last decade, DNA profiling based on microsatellite markers has deeply changed the way in which analysis of genetic diversity and cultivar genotyping are conducted in fruit crops and in grapes. Microsatellite markers have proven useful in parentage analysis [14,16-18] and in genetic characterization of cultivars [15,19], but di-nucleotide and a small number of tri-nucleotide repeats are the only ones currently in use in fruit crops and grapes. Standardization and exchange of information concerning grapevine genetic resources using reference microsatellite markers were already proposed in the past [15]. Recently, the list of di-nucleotide SSR markers, mainly developed by the Vitis Microsatellite Consortium, was extended [20].

In the present paper, a new set of microsatellite markers is proposed with the aim of minimizing genotyping errors. The new SSRs were retrieved from the whole genome sequence [21], allowing a wide search of long repeat motifs as candidate markers. The markers were ranked according to their information content, reproducibility, ease of scoring, and independent segregation.

## Methods

### Retrieving microsatellites from the grape genome sequence

Simple sequence repeats penta-, tetra-, and tri-nucleotides long, with a minimum number of 4, 5, and 6 tandem arrays of the core repeat, respectively, were retrieved from the grape sequence available at , using a modified version [9] of the software Sputnik, developed by Chris Abajian at the University of Washington .

Microsatellite sequences were selected from scaffolds anchored to the 19 linkage groups with the aim of selecting 38 well-distributed markers, ideally two for each linkage group, but not less than one. Different scaffolds were selected from the same linkage group to reduce the chance of selecting closely linked markers. Microsatellites located within or close to repetitive regions were discarded, which were identified with Repeat Marker, Tandem Repeats Finder, RepeatScout, and ReAS as described in [21]. Microsatellites with the highest number of core repeats were chosen and primer pairs were designed in their flanking regions using Primer3 [22]. Parameters were set up to have the same annealing temperature of 57°C for all primer pairs and a range of amplicon length as short as 80–200 bp to minimize the occurrence of insertion/deletions between the primer sites and the microsatellite repeat.

### PCR primer testing

Four genotypes, the *Vitis vinifera *cultivars Chardonnay and Cabernet Sauvignon, and the *Vitis *hybrids Bianca and 20/3, were used for a preliminary test of PCR amplification of the 94 markers initially selected. These grapes are the parents of two F1 populations available at the University of Udine [23], which were used for the analysis of segregation of the final set of markers (see below).

DNA was extracted from 0.1 g of young leaf tissue by means of the Qiagen DNeasy Plant Mini Kit, following the factory-recommended protocol.

PCR reactions were carried in 10 μL using 200 μM each dNTP, 0.2 μM each primer, 10 ng genomic DNA, and 0.2 U of HotMaster Taq polymerase (Eppendorf). The forward primers were labelled with 6-FAM or HEX fluorescent dyes. The PCR reactions were carried out in a PTC 200 thermal cycler (MJ Research) with the following thermal profile: one cycle at 95°C for 2 min, followed by 10 touch down cycles at 94°C for 20 s, 55°C – 0.5°C/cycle for 20 s, 65°C for 40s, followed by 15 cycles at 94°C for 20 s, 50°C for 20 s, 65°C for 40 s, and a final step of 1 hour at 65°C. PCR products were precipitated with 27.5 μl absolute ethanol and 1.0 μl 7.5 M ammonium acetate. Samples were washed twice with 70% absolute ethanol and re-suspended in 30–60 μl H_2_O.

One microliter of each PCR product was mixed with 0.1 μl Et400-R size standard (GE Health Care, USA), and 4.9 μl deionised H_2_O, centrifuged, denatured at 95°C for 2 min, cooled in ice, and separated on a MegaBACE 500 capillary sequencer (GE Health Care, USA).

Dye-labelled amplicons were automatically sized using internal standards and the Genetic Profiler v2.0 software (GE Health Care, USA) and then visually inspected.

### SSR polymorphism and quality check using different electrophoretic platforms

The primer pairs that gave successful amplifications in the previous step were used to screen 48 grapevine accessions, including both cultivars and rootstocks (See additional file 1 for the original data used to perform this analysis). DNA was extracted and PCR was performed as reported above. PCR products were diluted to equalize fluorescence intensity, added to 0.2 μL of LIZ 500 size standard, and separated by capillary electrophoresis using an ABI Prism 3730 DNA analyzer (Applied Biosystems). SSR were analyzed with Peak Scanner Software version 1.0 (Applied Biosystems).

Thirteen markers were randomly selected and their PCR products separated on a MegaBACE 500 capillary sequencer (GE Health Care, USA) with the protocol reported above. These data were used for comparing the absolute allele size, expressed as base pairs, obtained with different equipment, commercial size standards, and software.

### Data analysis

Data were processed using the software CERVUS, written by Tristan Marshall, available at the web site . Genotypes showing a single peak at a given locus were recorded as homozygous. CERVUS was used for the calculation of the number of alleles and their frequency, the observed and expected heterozygosity (*ho *and *he *respectively) [24], the presence of null alleles [25], the polymorphic information content (PIC) which measures the informativeness related to the expected heterozygosity [26], the fitting to Hardy Weinberg equilibrium, and the average non-exclusion probability, that is the probability that the genotypes at a single locus do not differ between two randomly-chosen individuals. The discrimination power at each locus (PD), which provides an estimate of the probability that two randomly sampled accessions of the study would be differentiated by their allelic profiles [27], was calculated using a Microsoft^® ^Excel spreadsheet.

### Creation of a SSRs ranking list

A ranking list of the best SSRs was produced in three steps. In the first step, the SSRs that did not pass either of the following conditions were discarded:

• amplification of a single locus, unless the second locus was clearly separated in size range from the first one

• negligible frequency of null alleles

• quality of the signal, i.e. sharp peak and low stuttering evaluated in a scale between 1 and 3, with 1 indicating good quality, 2 medium, and 3 low quality

In the second step, we evaluated the independent segregation of each pair of SSRs and discarded the markers with the lowest quality among those belonging to the same linkage group. For the third step see 'Genetic mapping', two paragraphs ahead.

### Allele sizing

Alleles of each locus were coded according to the number of their core repeats. The number of repeats in the allele of the sequenced genotype PN40024 was used as a reference and bins were created stepwise according to the core repeat length of any given locus. Alleles falling in between two bins were assigned the number of repeats of the lowest bin followed by a second number representing the estimated number of extra nucleotides. The allele coding is therefore represented by the number of repeats and the number of extra nucleotides separated by a dot (e.g. 6.3 means six repeats plus three extra nucleotides).

Ladders were constructed for three SSRs by selecting a set of cultivars that satisfied the following constrains: (a) to include all allelic variants of the population analysed in this study and (b) each allele being represented only once to have comparable signals of fluorescent intensity in the electrophoretic run. This was accomplished with LadderFinder, a software constructed *ad hoc *that tackled this issues as a maximal matching graph problem [28]. To compute the maximal matching, the Edmonds's non-bipartite matching algorithm was implemented. The software can be downloaded from the web site . The PCRs were carried out individually for each selected cultivar and the PCR products mixed in variable v:v ratios to obtain the most uniform height of peaks on the sequencer.

### Genetic mapping

Segregation analysis of the markers that passed the previous screening was conducted on two mapping populations, Chardonnay × Bianca and Cabernet Sauvignon × 20/3, as previously reported by Di Gaspero et al [23]. The segregation data of the new markers were integrated into the existing data set [23]. Parental maps were constructed using CARTHAGENE 0.999R [29]. Map position of each new marker was also assigned on the *Vitis *reference map of Doligez et al [30] by comparing the position of the pair of reference markers flanking a new marker in 'Chardonnay', 'Cabernet Sauvignon', 'Bianca', and/or '20/3' with the same reference markers placed on the integrated map.of Doligez et al [30]. The genetic interval where the marker was placed by map alignment was validated by referring any marker to its absolute chromosomal position in the 8.4 X genome assembly [21]. Map order was verified by a blastN search of the primer sequences of a new locus and its neighbour reference markers on the scaffolds of the 8X grape genome assembly . Segregation analysis was also used for disclosing the nature of some multi-locus markers, which had remained hidden by inspecting the 8.4 X genome assembly, and markers with null (non-amplified) alleles, which were both discarded.

## Results and discussion

### The selection of microsatellites

A total of 26,962 microsatellites carrying tri-, tetra-, and penta-nucleotide perfect repeats, with a minimum of 6, 5, and 4 core repeat units respectively were recovered from the 8.4 X grape genome sequence assembly. The microsatellites recovered for the three different classes were 15,934; 6,271; and 4,757, respectively. The distribution of microsatellites across the 19 chromosomes of PN40024 is reported in the additional file 2 (See additional file 2 for the original data used to perform this analysis). Some 69% of the microsatellites were located on scaffolds assigned to the chromosomes based on the 8.4 X assembly, and the position of the remainder is unknown (See additional file 2). This survey was limited to perfect microsatellites. Chromosome 14 showed the lowest number of microsatellites, one every 278 kb, considering all the three classes, and chromosome 9 the highest, with one every 183 kb (See additional file 3). The majority of the microsatellites consisted of poly AAT, AAAT, and AAAAT motifs for the three classes of microsatellites, respectively (data not shown).

Ninety-four sequences were selected based on the following criteria: length of the microsatellite region, presence of flanking sequences appropriate for primers design, and absence of single-base stretches or other low complexity regions upstream and downstream of the repeated motif. Different motifs were retrieved to test their performance and minimise the typical drawbacks of microsatellite DNA amplification, such as amplification of ghost bands or stuttering peaks.

Of the 94 selected sequences, 29 contained tri-nucleotide perfect motifs, 45 contained tetra-nucleotide perfect motifs, and 20 contained penta-nucleotide perfect repeats, respectively.

### Testing of the primer pairs

Amplification was carried out on the four parents of the two mapping populations and yielded amplicons with all 94 primers pairs tested. Fifty one of these met the criteria needed to enter the next step of analysis. The remaining 46% were discarded because they showed either one or a combination of severe drawbacks, such as weak amplification (4%), high stuttering (6%), unreadable multi- peak profiles (23%), and lack of polymorphism (13%). This is not unexpected considering that the same PCR protocol was applied to all primers pairs, for the sake of uniformity. Some of these drawbacks could be eliminated using specific PCR conditions, optimised for each primer pair, though that contrasted with the aim of this work.

### Evaluation of SSR quality and polymorphism

After the analysis was extended to 48 genotypes, six additional markers were excluded because of the presence of multiple peaks in some varieties of the extended panel or the amplification of double peaks at 1 bp interval that hamper a clear distinction between true alleles and stuttering.

The remaining 45 SSRs were kept and ranked according to their quality (See additional file 3 for the original data used to perform this analysis). The 26 markers with the highest quality score had no technical drawbacks, such as presence of stuttering or amplification of extra peaks. The number of alleles ranged from two to sixteen, the observed and expected heterozygosity ranged from 0.124 to 0.565 and from 0.099 to 0.617 respectively, and the PIC ranged from 0.093 to 0.604 (See additional file 4 for the original data used to perform this analysis). The non-exclusion probability between two unrelated individuals (NE-I) and between two hypothetical full siblings (NE-SI) ranged from 0.025 to 0.517 and 0.219 to 0.601, respectively. These values define the probability that the genotypes at a single locus do not differ between two randomly-chosen individuals. This probability may be calculated in two ways. The basic formula assumes that the two individuals are unrelated, while a more conservative formula assumes the two individuals to be full sibs [31].

Null alleles are a common cause of apparent deviations from Hardy-Weinberg equilibrium at microsatellite loci [32] and may interfere with pedigree reconstructions. In the absence of a null allele, the estimated frequency of null alleles would be close to zero (See additional file 4), and may be slightly negative in the presence of an excess of observed heterozygous genotypes. A locus with a large positive estimate of null allele frequency indicates an excess of homozygotes but does not necessarily imply that a null allele is present. For this reason, the presence of null alleles were also investigated by segregation analysis (see below).

Twenty seven markers (60%) showed a regular step, with a ± 0.5 bp tolerance in size variation within an allelic bin, following a stepwise mutation model. Sequencing of all the alleles for each locus should be performed to confirm that length variation is due to different number of core repeats rather than to other insertion or deletions in the flanking sequences. Sequencing should also confirm the real size of each allele. Four loci (9%), namely VChr10a, VChr13c, VChr17b, and VChr18d, showed a perfect stepwise variation but only the first one was scored as a quality 1 marker.

**Figure 1:**
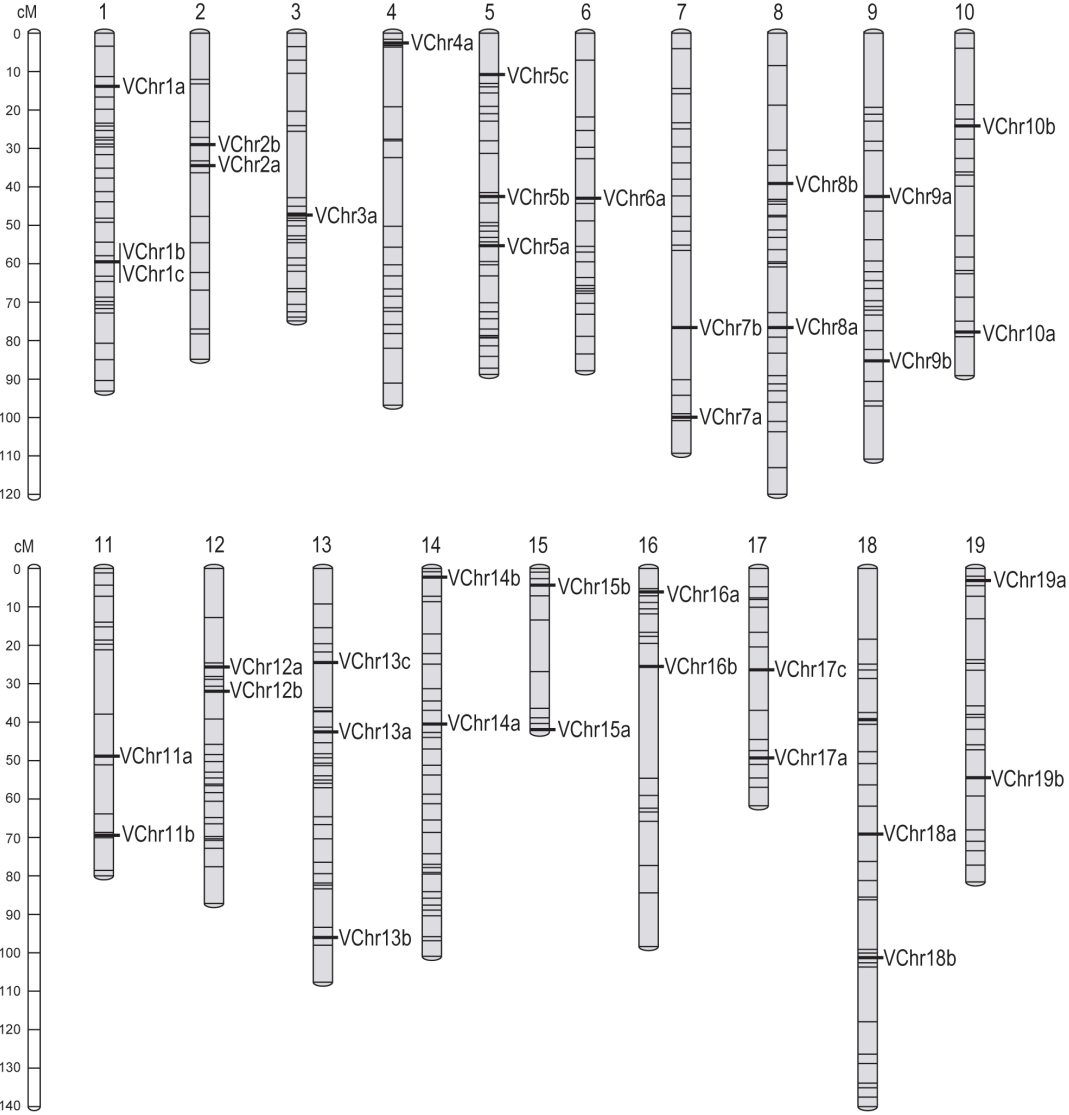
**Diagram of the 19 grapevine chromosomes showing the distribution of the 38 SSR markers (*thick lines*) proposed for grape fingerprinting**. Map position of markers of the *Vitis *reference map (*thin lines*) and their relative distances were drawn according to Doligez et al. (2006) [30]. Map distances are scaled to the reference bar on the left of the figure and expressed in cM Kosambi.

A total of 38 markers were finally selected by combining quality score, power of discrimination (PD), and whole genome distribution (1–3 markers/chromosome) (Table 1; Figure 1). All 38 markers are of quality score 1 or 2. All 19 linkage groups but one, LG 4, were covered by at least one of the 26 top quality markers. With the present set of markers, only the LGs 3, 4, and 6 have only one marker each (Figure 1).

**Table 1:** List of 38 SSR markers ranked according to their PD (power of discrimination).

Primer	LG	PD	N of genotypes	min genotypic frequency	max genotypic frequency	Quality score
**VChr8b**	**8**	**0,956**	**30**	**0,021**	**0,083**	**2**

**VChr3a**	**3**	**0,933**	**27**	**0,021**	**0,188**	**1**

*VChr8a*	*8*	*0,926*	*23*	*0,021*	*0,149*	*1*

**VChr9a**	**9**	**0,925**	**18**	**0,021**	**0,149**	**1**

*VChr9b*	*9*	*0,905*	*16*	*0,021*	*0,188*	*2*

**VChr19a**	**19**	**0,902**	**20**	**0,021**	**0,167**	**1**

**VChr5b**	**5**	**0,899**	**17**	**0,021**	**0,167**	**1**

**VChr15b**	**15**	**0,898**	**17**	**0,021**	**0,188**	**2**

*VChr5c*	*5*	*0,895*	*13*	*0,021*	*0,167*	*1*

**VChr11b**	**11**	**0,874**	**11**	**0,024**	**0,214**	**2**

**VChr7b**	**7**	**0,866**	**12**	**0,021**	**0,188**	**2**

**VChr13a**	**13**	**0,855**	**13**	**0,021**	**0,292**	**1**

*VChr13c*	*13*	*0,854*	*11*	*0,021*	*0,250*	*2*

**VChr18a**	**18**	**0,852**	**15**	**0,021**	**0,319**	**1**

*VChr19b*	*19*	*0,852*	*11*	*0,021*	*0,250*	*1*

**VChr14b**	**14**	**0,834**	**19**	**0,021**	**0,362**	**2**

*VChr15a*	*15*	*0,826*	*13*	*0,022*	*0,326*	*1*

**VChr1b**	**1**	**0,821**	**10**	**0,021**	**0,292**	**1**

*VChr18b*	*18*	*0,819*	*9*	*0,021*	*0,271*	*1*

**VChr12a**	**12**	**0,814**	**12**	**0,021**	**0,313**	**1**

**VChr10b**	**10**	**0,798**	**8**	**0,021**	**0,333**	**2**

**VChr16a**	**16**	**0,796**	**12**	**0,021**	**0,375**	**1**

**VChr4a**	**4**	**0,787**	**12**	**0,021**	**0,333**	**2**

*VChr13b*	*13*	*0,782*	*15*	*0,021*	*0,417*	*2*

*VChr10a*	*10*	*0,757*	*15*	*0,021*	*0,458*	*1*

**VChr6a**	**6**	**0,733**	**7**	**0,043**	**0,362**	**1**

*VChr11a*	*11*	*0,733*	*9*	*0,021*	*0,438*	*1*

*VChr1a*	*1*	*0,721*	*14*	*0,021*	*0,500*	*1*

**VChr2b**	**2**	**0,714**	**7**	**0,021**	**0,417**	**2**

*VChr16b*	*16*	*0,711*	*10*	*0,021*	*0,479*	*1*

*VChr14a*	*14*	*0,709*	*5*	*0,021*	*0,375*	*1*

*VChr5a*	*5*	*0,700*	*9*	*0,021*	*0,500*	*1*

*VChr7a*	*7*	*0,665*	*4*	*0,021*	*0,396*	*1*

*VChr12b*	*12*	*0,601*	*3*	*0,208*	*0,542*	*2*

*VChr1c*	*1*	*0,598*	*4*	*0,021*	*0,500*	*1*

**VChr17a**	**17**	**0,585**	**4**	**0,022**	**0,578**	**1**

*VChr2a*	*2*	*0,518*	*3*	*0,021*	*0,521*	*1*

*VChr17b*	*17*	*0,160*	*4*	*0,021*	*0,915*	*1*

In bold are reported the 19 SSR markers with the highest PD for each chromosome of the grape genome, in italics the other 19 SSR markers.

N of genotypes report the number of genotypes recognized with each marker Min and max genotypic frequency report the frequency of the genotype less and more represented respectively

Some 52% of the markers have a PD higher then 0.8 and only 10% have a PD lower then 0.5 and were included the in present work because less polymorphic loci have lower mutation rates and are useful for parentage testing [4].

The power of discrimination of the six most used markers in current grape fingerprinting, VVMD5, VVMD7, VVMD27, VVS2, VrZAG62, and VrZAG79 [15] was calculated from data available from different public sources (; ; and ) for the 48 samples used in the present work. The PD values ranged between 0.93 and 0.97 for the markers VVMD27 and VVS2 respectively. The six markers with the highest PD, among those presented in the present work, ranged between 0.90 and 0.96 (Table 1). From this point of view, the information content and the power of discrimination of the two sets of SSRs are similar.

**Figure 2:**
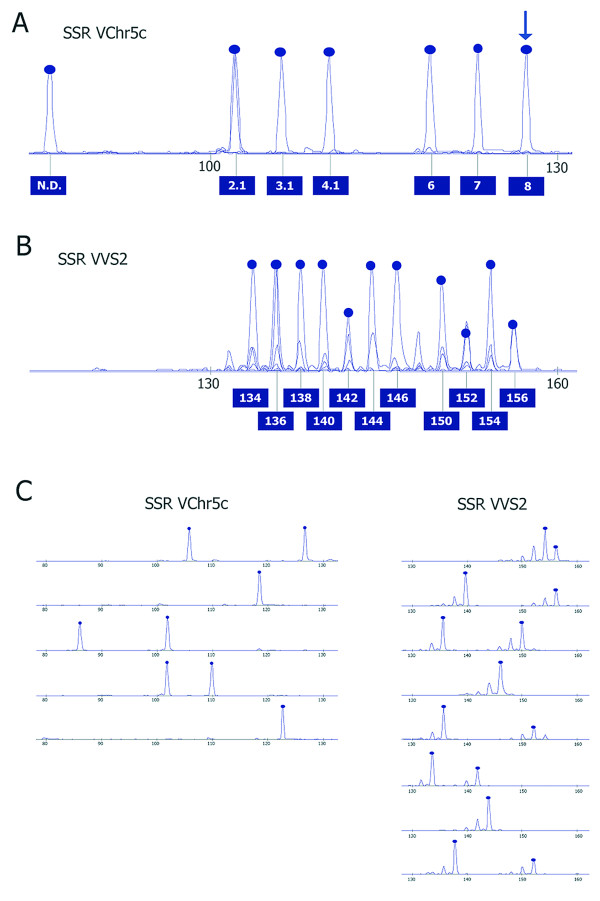
**Electrophoretic pattern of true alleles (peaks with *blue dots *on top) and stuttering peaks of a tetra-nucleotide microsatellite (VChr5c) identified in this work and a di-nucleotide microsatellite (VVS2) currently used for grapevine fingerprinting**[15]. Panel A and B report for each marker the composite pattern of all alleles found in the population of 48 cultivars of this study, obtained by graphical overlap of individual patterns of panel C. *Blue arrow *points to the homozygous allele in the sequenced genome of PN40024 for VChr5c, whose number of core repeats (8) is used for coding all other alleles accordingly, as described in the text. The allele coded N.D. is likely generated by a deletion in the sequence intervening the primer sites. Panel C reports the individual electropherograms of 4 and 8 cultivars which cumulatively represent all of the alleles for VChr5c and VVS2, respectively, found in a set of 48 cultivars.

The electrophoretic patterns of true alleles and stuttering peaks of a tetra-nucleotide microsatellite identified in this work and a di-nucleotide microsatellite currently used for grapevine fingerprinting [15] were compared and are shown in Figure 2 as an example. This illustrates the main differences between the two kinds of SSRs from a technical point of view. Di-nucleotide microsatellite markers usually have a high number of alleles, with a frequent 2-bp allelic incremental step, which results in the peaks of true alleles overlapping the stuttering peaks of the closest alleles. Microsatellites with longer core motifs have a lower number of alleles, peak distances are larger, and stuttering peaks are attenuated, which all render the scoring of microsatellites with long core repeats more reliable (Figure 2).

By combining segregation analysis in two mapping populations and marker order validation on the 8.4X genome assembly, all 38 markers were positioned on the *Vitis *reference genetic map (Figure 1). All markers were heterozygous in at least one parent of the mapping populations analysed. Sixteen and 13 markers were heterozygous in three and four parents, respectively. Seven and two markers were heterozygous in only two or one of the parents, respectively. Segregation analysis unveiled the presence of additional heterozygosity only for the markers VChr17a and VChr5a, due to the presence of null alleles not amplified in seemingly homozygous parents.

Pairs or triplets of markers within each chromosome were in general not clustered. Only markers on LGs 1, 2, and 12 were linked at less than 10 cM. Markers VChr1a and VChr1b on LG1 co-segregated and their physical distance was approximately 50 kb in PN40024. However, the occurrence of loci on the same chromosome that are only 1 million or less of base pairs apart can be shuffled separately because of recombination hot spots and patterns of linkage disequilibrium as demonstrated in hundreds of human population studies [4]. The marker VChr10b mapped in the same position as the already published marker VMC4f9-1. The match of the corresponding primer sequences on the PN40024 assembly confirmed they amplified the same locus. Three markers amplified more than two alleles per genotype, in at least one parent. Marker VChr6a amplified alleles of similar size from two loci, which were located 12 kb away from each other, based on the PN40024 sequence. One locus was perfectly matched by the primer pair VChr6a, the second one had one mismatch per primer. Two more markers (VChr3a and VChr15a) amplified more than two alleles per genotype, but the corresponding primers matched a single locus in the PN40024 sequence. This could have occurred either because these loci are duplicated in some grape genotypes but not in PN40024, because the second locus was not present in the assembled fraction of the PN40024 genome, or because the additional alleles were amplified from mutated cell layers, as sometimes happens in meristematic tissues of vegetatively propagated grapevines [33]. In contrast, marker VChr8b was duplicated within 1 kb in the PN40024 genome with no mismatches over the primer sequences, but only one locus was evident in the segregation analysis. The second locus might be undetected in the parents and in the progeny either because of alleles of overlapping size or due to a much longer allele size, given the expected size of the second locus in PN40024 was ~1700 bp, and therefore too long to be either amplified and detected.

Irrespectively to the genome localisation, markers with the highest PD and quality score were considered useful for cultivar fingerprinting, pedigree analysis, and parentage testing. As an example, the 48 grape cultivars and rootstocks were differentiated using only two markers with the highest PD value (VChr3a, VChr8a) with the only exception, represented by the two possible synonymous cultivars Cannonau and Tocai Rosso (data not shown).

The combined non-exclusion probability, between unrelated or full sibling individuals, considering the 18 best quality markers for each linkage group, were 1.23 × 10^-15 ^and 8.6 × 10^-7 ^respectively. These values are appreciably low, considering the low number of varieties of the panel.

### Allele sizing

Electrophoretic separation was performed using two different platforms the ABI Prism 3730 DNA analyzer (Applied Biosystems) and the MegaBACE 500 capillary sequencer (GE Healthcare Biosciences, USA) with their associated Peak Scanner Software version 1.0 and Genetic Profiler v2.0 software, respectively. The allele sizes calculated by Genetic Profiler were usually larger then those assigned by the Peak Scanner Software of the Applied Biosystems platform (min 0.0 bp; max 3.8 bp). These differences were expected according to the literature and could be mainly due, in order of importance, to the size standard, the polymers used to fill the capillaries, the machine type, and the run conditions [6].

These discrepancies can not be eliminated and for this reason the protocol for human fingerprinting recommends the use of allelic ladders.

**Figure 3:**
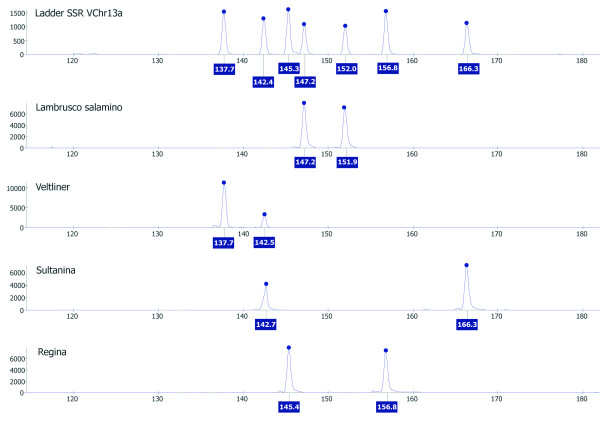
**Example of allelic ladder developed for the marker VChr13a**. The first electropherogram reports the ladder produced by mixing amplicons from the individual amplification of DNA from four cultivars, whose individual electropherograms are reported below and whose alleles cumulatively represent all alleles found in the population of this study. The sizes of the alleles are expressed in bp. Electrophoresis and analysis were performed on a MegaBace 500 Sequencer analyzer (GE HealthCare) using the ET-400-R size standard.

In order to avoid these discrepancies that could lead to difficulties in comparing data between different laboratories, an example of an allelic ladder was produced for the markers VChr13a, VChr9a, and VChr8a, showing 7, 8, and 12 alleles respectively. A marker ladder carries all or most known alleles that exist in a population and an allele in a genotype is assigned by comparison of the distance (bp) of its peak from the closest ladder peaks rather than by absolute sizing of the amplicon. An example of allelic ladder for the marker VChr13a is reported in Figure 3. Given that tri-, tetra-, and penta-nucleotide SSR markers are less prone to stuttering and the space between adjacent alleles is larger than in di-nucleotide SSRs, mis-assignation of peaks to the true allele would be less likely with the new markers than with the di-nucleotide markers currently in use. However, the development of allelic ladders for all the loci is still in progress. Meanwhile, sizes were assigned to all of the alleles found in the present work by considering the number of core repeats counted in the allele of the homozygous PN40024. From its genome sequence, the number of core repeats was counted and associated with the allele length obtained by genotyping the same DNA. For example, the size assigned to PN40024 at the *locus *VChr1a, was 7 due to the presence of seven ATCC repeats, then the other alleles found in the population are expected to differ for one or more units of the core 4 bp repeat (See additional file 5 for the original data used to perform this analysis). Not at all alleles were assigned an integer number because the sizes obtained from the genotyping could be affected by additional insertions or deletions of one or more bases, inside or outside of the microsatellite repeat. For example, the *locus *VChr1a had nine alleles with the following assigned sizes: N.D., 3.1, 5, 6, 7, 8.1, 9.1, 11.1, and 12.1. No size was assigned to the shortest allele (N.D.) because the number of calculated repetitions would be less then zero, probably due to a large deletion. The other alleles could have an integer number (5, 6, or 7) or a decimal number (3.1, 8.1, etc.) because of an extra base insertion. To conclude on this point, fingerprinting analysis based on SSRs relies on the estimate of amplicon length, and differences due to homoplasy, that is alleles of the same size and different nucleotide sequence, are usually not considered.

## Conclusion

The available grape genome sequence  enabled the recovery of thousands of perfect microsatellite markers with long repeats, namely penta-, tetra-, and tri-nucleotides, which are the most recommended markers for individual genotyping, allowing correct binning and sizing of alleles, and thus the construction of robust databases of individual profiles.

We have selected 38 of these which show high polymorphism, easy separation of alleles, and reproducibility of analysis, as well as genome coverage across all chromosomes and a lack of drawbacks such as amplification of multiple loci, high frequency of null alleles, and other problematic features.

Of these, nineteen, distributed one per chromosome and ranked according to their information content, were proposed in a first list and recommended for fingerprinting. The combined non-exclusion probability for unrelated and full sib individuals was 1.23 × 10^-15 ^and 8.6 × 10^-7^, respectively. We expect that, as more and more profiles of varieties accumulate in databases, a more accurate estimate of allele frequencies and a more comprehensive sampling of rare alleles will make kinship analysis in grape very robust and reliable.

A consistent genotyping protocol should include different procedures: (a) the use of microsatellites with long repeat motifs; (b) the use of capillary electrophoresis to separate the PCR amplicons; (c) the analysis of data with software that uses binning algorithms to call and size alleles [34,35,4]; (d) the use of specific allelic ladders for each SSR, and if this is not possible, the use of one or more reference cultivars of known profile side by side to the sample of study [15]. Many of these advantageous practices have been adopted in the present study.

It is likely that the use of 19 markers exceeds the practical need for many identity and pedigree analyses. For this, markers are prioritised so that, whatever the number of markers adopted, there is a minimum set of markers for which the profiles are comparable. In other words, if one laboratory uses eight primers, another lab uses twelve, and another eighteen, there are eight markers common to all three labs and twelve common to the last two labs. Grape varieties selected in Western Europe, which account for most of the worldwide production of wine, likely have extensive coancestry [36,20], that is common origin from the hybridisation of a few ancestors. Because of this, using too few markers for fingerprinting could hamper the discrimination of sibling varieties. For this reason we recommend using at least the first 19 markers of the list.

The grapevine industry relies on many somatic mutants of many standard cultivars. This genetic variation within each cultivar raises some concern about how to identify such variants. Some SSR markers have occasionally showed allelic variation among clones of the same variety, and this should be carefully taken into account in forensics and parentage reconstruction [33]. The more general problem of accurately discriminating among clonal variants within a given cultivar requires different approaches. One option could be the use of high throughput techniques, such as arrayed SNPs; another could consider the short reads produced with new sequencing technologies, which both allow scanning a larger part of a genome. One might also consider variations due to transposable elements or explore variations in DNA methylation patterns.

## Authors' contributions

GC, RT, and MM conceived and designed the fingerprinting experiments. GDG and AP carried out the mapping analysis. MTM performed experimental work on microsatellite characterization and cultivar fingerprinting. GC performed the statistical analysis and wrote the manuscript to which all authors contributed, and read and approved the final manuscript.

## Supplementary Material

Additional file 1 **List of cultivars and hybrids used for the evaluation of the grape SSR**. Click here for file

Additional file 2 **Microsatellite distribution among the 19 chromosomes of the grape genome homozigous line PN40024**. Click here for file

Additional file 3 **List of 45 tri-, tetra- and penta-SSR markers developed in grape ranked according to their linkage group and quality score**. Click here for file

Additional file 4 **Summary of statistics for the 45 SSR markers developed in grape**. Click here for file

Additional file 5 **List of the alleles found in the fingerprinting of 48 grape cultivars and rootstocks of the study**. Click here for file
