# Supplementary material for: A set of microsatellite markers with long core repeat optimized for grape (Vitis spp.) genotyping
Source: BMC Plant Biol. 2008 Dec 16;8:127. doi: 10.1186/1471-2229-8-127 (PMC2625351; doi:10.1186/1471-2229-8-127)
Supplement: Additional file 1 — List of cultivars and hybrids used for the evaluation of the grape SSR. [file 1471-2229-8-127-S1.doc]

Additional file 1: List of cultivars and hybrids used for the evaluation of the grape SSR.

| n | sample | clone ^(1)^ | Origin | Species |
| --- | --- | --- | --- | --- |
| 1 | Chardonnay | VCR 4 | France | *V. vinifera* |
| 2 | Sangiovese | VCR 16 | Italy | *V. vinifera* |
| 3 | Malvasia Bianca Lunga | VCR 10 | Italy | *V. vinifera* |
| 4 | Aglianico | VCR 7 | Italy | *V. vinifera* |
| 5 | Negro Amaro | VCR 10 | Italy | *V. vinifera* |
| 6 | Pinot Blanc | VCR 1 | France | *V. vinifera* |
| 7 | Raboso Veronese | VCR 3 | Italy | *V. vinifera* |
| 8 | Riesling Blanc | VCR 3 | Germany | *V. vinifera* |
| 9 | Cabernet Franc | VCR 10 | France | *V. vinifera* |
| 10 | Grechetto | VCR 2 | Italy | *V. vinifera* |
| 11 | Lambrusco Salamino | VCR 1 | Italy | *V. vinifera* |
| 12 | Tocai Friulano | VCR 9 | Italy | *V. vinifera* |
| 13 | Tocai Rosso | VCR 3 | Italy | *V. vinifera* |
| 14 | Uva di Troia | VCR 1 | Italy | *V. vinifera* |
| 15 | Vermentino | VCR 1 | Italy | *V. vinifera* |
| 16 | Cardinal | ISV-VCR 26 | USA | *V. vinifera* |
| 17 | Albana | VCR 21 | Italy | *V. vinifera* |
| 18 | Moscato Giallo | VCR 5 | Italy | *V. vinifera* |
| 19 | Moscato Bianco | VCR 3 | Italy | *V. vinifera* |
| 20 | Trebbiano Toscano | VCR 8 | Italy | *V. vinifera* |
| 21 | Muller Thurgau | VCR 1 | Switzerland | *V. vinifera* |
| 22 | Lambrusco Maestri | VCR 1 | Italy | *V. vinifera* |
| 23 | Traminer | VCR 6 | France | *V. vinifera* |
| 24 | 420 A | VCR 103 | - | Hybrid rootstock |
| 25 | Kober 5 BB | VCR 102 | - | Hybrid rootstock |
| 26 | SO4 | VCR 105 | - | Hybrid rootstock |
| 27 | 161-49 | VCR 112 | - | Hybrid rootstock |
| 28 | 1103 P | VCR 107 | - | Hybrid rootstock |
| 29 | Merlot | VCR 494 | France | *V. vinifera* |
| 30 | Corvina Veronese | VCR 448 | Italy | *V. vinifera* |
| 31 | Barbera | VCR 433 | Italy | *V. vinifera* |
| 32 | Verdicchio | VCR 3 | Italy | *V. vinifera* |
| 33 | Gamay | VCR 1 | France | *V. vinifera* |
| 34 | Prosecco | ISV 3 | Italy | *V. vinifera* |
| 35 | Sultanina | VCR 122 | Turkey | *V. vinifera* |
| 36 | Chrupka Cervena | VCR 216 | Czech Republic | *V. vinifera* |
| 37 | Pannonia Kinsce | VCR 220 | Hungary | *V. vinifera* |
| 38 | Sylvaner Verde | VCR 186 | Austria | *V. vinifera* |
| 39 | Veltliner | VCR 233 | Germany | *V. vinifera* |
| 40 | Regina | ISV 6 | Italy | *V. vinifera* |
| 41 | Greco di Tufo | VCR 11 | Italy | *V. vinifera* |
| 42 | Carmenere | VCR 22 | France | *V. vinifera* |
| 43 | Syraz | 16/54 | France | *V. vinifera* |
| 44 | Canaiolo | VCR 10 | Italy | *V. vinifera* |
| 45 | Tempranillo | - | Spain | *V. vinifera* |
| 46 | Garganega | VCR 13 | Italy | *V. vinifera* |
| 47 | Schiava Grossa | VCR 12 | Italy | *V. vinifera* |
| 48 | Cannonau | VCR 23 | Italy | *V. vinifera* |

1. VCR = clones developed by Vivai Cooperativi Rauscedo – Italy;

ISV = clones from Istituto Sperimentale per la Viticoltura – Conegliano _ Italy
