# Supplementary material for: A set of microsatellite markers with long core repeat optimized for grape (Vitis spp.) genotyping
Source: BMC Plant Biol. 2008 Dec 16;8:127. doi: 10.1186/1471-2229-8-127 (PMC2625351; doi:10.1186/1471-2229-8-127)
Supplement: Additional file 2 — Microsatellite distribution among the 19 chromosomes of the grape genome homozigous line PN40024. [file 1471-2229-8-127-S2.doc]

Additional file 2: Microsatellite distribution among the 19 chromosomes of the grape genome homozigous line PN40024. Tri, tetra and penta-nucleotide repeats, with a minimum of 6, 5 and 4 core repeat units respectively were recovered from the 8.4 X grape genome sequence assembly.

| Chromosome number | Size (bp) | Pentanucleotide repeats | Frequency of pentanucleotide repeats | Tetranucleotide repeats | Frequency of tetranucleotide repeats | Trinucleotide repeats | Frequency of trinucleotide repeats | Sum of the frequency of the three classes |
| --- | --- | --- | --- | --- | --- | --- | --- | --- |
| chr1 | 21,127,006 | 205 | 103,059 | 263 | 80,331 | 713 | 29,631 | 213,021 |
| chr2 | 17,664,209 | 178 | 99,237 | 219 | 80,658 | 579 | 30,508 | 210,404 |
| chr3 | 11,530,193 | 100 | 115,302 | 143 | 80,631 | 382 | 30,184 | 226,116 |
| chr4 | 19,293,076 | 213 | 90,578 | 220 | 87,696 | 560 | 34,452 | 212,726 |
| chr5 | 23,428,299 | 213 | 109,992 | 304 | 77,067 | 720 | 32,539 | 219,598 |
| chr6 | 24,148,918 | 249 | 96,984 | 299 | 80,766 | 791 | 30,530 | 208,279 |
| chr7 | 15,409,890 | 137 | 112,481 | 216 | 71,342 | 441 | 34,943 | 218,766 |
| chr8 | 21,569,352 | 198 | 108,936 | 305 | 70,719 | 634 | 34,021 | 213,676 |
| chr9 | 16,532,244 | 188 | 87,937 | 247 | 66,932 | 594 | 27,832 | 182,702 |
| chr10 | 11,853,394 | 119 | 99,608 | 130 | 91,180 | 339 | 34,966 | 225,754 |
| chr11 | 15,894,710 | 159 | 99,967 | 188 | 84,546 | 568 | 27,984 | 212,497 |
| chr12 | 21,367,224 | 201 | 106,305 | 290 | 73,680 | 688 | 31,057 | 211,042 |
| chr13 | 16,772,351 | 166 | 101,038 | 215 | 78,011 | 560 | 29,951 | 209,000 |
| chr14 | 24,912,860 | 182 | 136,884 | 247 | 100,862 | 617 | 40,377 | 278,123 |
| chr15 | 11,991,189 | 130 | 92,240 | 181 | 66,250 | 442 | 27,129 | 185,619 |
| chr16 | 12,683,262 | 141 | 89,952 | 174 | 72,892 | 456 | 27,814 | 190,659 |
| chr17 | 14,822,103 | 154 | 96,247 | 194 | 76,403 | 547 | 27,097 | 199,747 |
| chr18 | 25,640,441 | 253 | 101,346 | 328 | 78,172 | 751 | 34,142 | 213,659 |
| chr19 | 15,984,336 | 161 | 99,282 | 223 | 71,679 | 566 | 28,241 | 199,201 |
|  | 342,625,057 | 3,347 | 102,368 | 4,386 | 78,118 | 10,948 | 31,296 | 211,781 |

The columns of the frequencies of each motif type give an estimate of the average distance between two microsatellites in base pairs.
