# Supplementary material for: A set of microsatellite markers with long core repeat optimized for grape (Vitis spp.) genotyping
Source: BMC Plant Biol. 2008 Dec 16;8:127. doi: 10.1186/1471-2229-8-127 (PMC2625351; doi:10.1186/1471-2229-8-127)
Supplement: Additional file 3 — List of 45 tri-, tetra- and penta-SSR markers developed in grape ranked according to their linkage group and quality score. [file 1471-2229-8-127-S3.doc]

Additional file 3: List of 45 tri-, tetra- and penta-SSR markers developed in grape ranked according to their linkage group and quality score.

| Primer name | forward sequence | reverse sequence | length (bp) of the sequenced allele | motif | LG | N of alleles | Range | Quality score |
| --- | --- | --- | --- | --- | --- | --- | --- | --- |
| VChr1a | TTCATACCTTGCAGGGAGCTA | TGATTTCCATTCCCAAATTCA | 219 | ATCC | 1 | 9 | 175-244 | 1 |
| VChr1b | AGATGGGTGGCATTAGCAAG | TTATTTCCCTCCCTCGCTGT | 112 | ATCC | 1 | 6 | 90-111 | 1 |
| VChr1c | CTGGCCTTATGCACAAAGTG | GATGAACACATCAATCAAATACCC | 100 | AGCC | 1 | 3 | 87-100 | 1 |
| VChr2a | GGTCCGCTTTTGAGAAGAAA | CATGTGAACGCGCTAAACAC | 146 | AGGC | 2 | 3 | 137-155 | 1 |
| VChr2b | CCTCCTGCGAACAAGTCTGT | GTTGCTGGATTTGTGGAAGG | 123 | AGCT | 2 | 6 | 112-128 | 2 |
| VChr2c | CTCAAAGCCCTCCAATTCAA | GGGCTCATGTGTCTGGAGTT | 149 | AGCC | 2 | 5 | 147-158 | 2 |
| VChr3a | CAATCATATGAGCAAGGCATGT | GCTTCCTGAAATTTGTGTCCA | 199 | AAT | 3 | 14 | 175-249 | 1 |
| VChr4a | CAACTGGGATCCAAGACCTC | CAGCTTCACAGGTAACCACA | 197 | AAAG | 4 | 7 | 173-203 | 2 |
| VChr5a | ACTTGGCGAGTATTTGTTCTAAA | CCGCTTTGTGAAGGTATCCA | 198 | AGATG | 5 | 11 | 183-259 | 1 |
| VChr5b | CTTCTCGGTCATGGTCATTG | CTCCTTCCACCTCTGGTTCA | 198 | AAAG | 5 | 10 | 179-219 | 1 |
| VChr5c | CCCATCAGTTTGCCTATGAA | TTTGATCTTGTTATTGTGCTGTTAC | 127 | ACAT | 5 | 7 | 83-123 | 1 |
| VChr6a | AATGTTGAGCTTTGGGCTTG | CCAATTCTTCCATACCTCAAAA | 184 | AATC | 6 | 4 | 173-180 | 1 |
| VChr7a | TCCGTGTCACAAAGAACATGA | ATTAGGGCACTGCCTCTTCC | 144 | AAAAG | 7 | 3 | 126-140 | 1 |
| VChr7b | AAAGGGCCTAAACTCTTAATAACTTG | TGCTTTATAGACACTAACCCACAAA | 188 | ACAT | 7 | 6 | 172-195 | 2 |
| VChr7c | CACTTCTCTGCCACCCATTT | GGTTGGAAATTCTAGGGCATT | 112 | ATGC | 7 | 3 | 101-108 | 2 |
| VChr8a | ACCCACTGCCACTCTCTCAT | AAATCTCCGGGATCCTTTTG | 172 | AAT | 8 | 12 | 172-206 | 1 |
| VChr8b | TGTGTGATGTTTTGTCGATGG | TGAACCAAGTTCTAATTTACATTTCC | 142 | AAG | 8 | 16 | 58-156 | 2 |
| VChr9a | GCGACAGCATCACTTCAATC | GAATTGCCAAGGACAAGGAG | 114 | AAG | 9 | 8 | 87-117 | 1 |
| VChr9b | AGCGTCATGACAGGTATCAGAA | AAAGAATTAATCATTACCATTTCACG | 161 | AAT | 9 | 10 | 102-160 | 2 |
| VChr10a | AAATGTTTAGTAGCCTCATTTTGTTT | TTTGTTCGGAACTACTCTTCTTCA | 136 | ACT | 10 | 8 | 98-137 | 1 |
| VChr10b | CCATGTCCAACCGAAACAAC | CAGAAATCTCGTGTCGCTCA | 140 | AAC | 10 | 5 | 116-136 | 2 |
| VChr11a | GGGATAAGGTGAAAGCCTCA | ATGCTTGGTATCTGGCAACC | 197 | AAAG | 11 | 6 | 178-207 | 1 |
| VChr11b | TGAGTTGAGCTATTGGCTTTGA | AGCAACTCTGTCCATCCATGT | 163 | AGAT | 11 | 5 | 151-163 | 2 |
| VChr12a | GCTTTAAATGTTAGATTAGGGCACTC | TCCATGTTGTTTGCTCTTTCC | 136 | AATT | 12 | 7 | 126-146 | 1 |
| VChr12b | AAACACAAGGTTGCATTGGA | GGCTTTCTTGTGGACTTAAATGA | 170 | AATT | 12 | 2 | 161-169 | 2 |
| VChr13a | TGGCAGAGCAAATGAATCAA | TTGGATGGATTGGAATGACC | 155 | AAAAG | 13 | 7 | 135-165 | 1 |
| VChr13b | TAAGCATTCTGGGCTTTTCC | TCGTCTATATGCGACCTTGG | 156 | AAAT | 13 | 8 | 145-170 | 2 |
| VChr13c | AGACCCAAGGGCAAGGTACT | AACACCGTTAGGCATACTCCA | 137 | AAT | 13 | 5 | 114-135 | 2 |
| VChr13d | AATCTGACGCCATGAGGAAG | TCGTCTATATGCGACCTTGG | 175 | AATC | 13 | 4 | 174-191 | 3 |
| VChr14a | AACCTGGGATGCTGAGAATG | TGCATGCATATGGATCTTGT | 133 | AATC | 14 | 3 | 128-189 | 1 |
| VChr14b | CAATTGAACACTTACACTCACAATCA | TGTGACTAAAGGTTATTAGCAGGA | 195 | ATC | 14 | 15 | 176-243 | 2 |
| VChr15a | CAATCCCAACAGTTCCATGA | CGTTTTCTCCTTCGGACAAG | 151 | ATCC | 15 | 8 | 127-165 | 1 |
| VChr15b | GGGTCCAATTCCTTTTGGTT | CGAAAGACTCAATTGCCACA | 124 | AAT | 15 | 10 | 90-151 | 2 |
| VChr16a | TTCATGTGTGACACCCCTTT | AATGTCCATGCTTCAAAATACC | 162 | AAAT | 16 | 8 | 100-167 | 1 |
| VChr16b | ATAAGGCGCTGACTTTGTGA | CCAGGAGATCAACCACCATT | 189 | AATT | 16 | 7 | 165-193 | 1 |
| VChr16c | TTTCAATATTCCAAATGTGACCT | CATTTCTTTGCTCTTCCTGCT | 160 | AATT | 16 | 4 | 151-161 | 2 |
| VChr17a | AGGAAGAGGATTGATCACCA | GTGCCAACCCTTGCACTATT | 187 | AACC | 17 | 3 | 170-184 | 1 |
| VChr17b | CCAAAGCCGACAACTTCTTC | CCGCCATAAACCCTAAACCT | 162 | ACTC | 17 | 3 | 154-162 | 1 |
| VChr17c | CCATGTTCCATCCCACTTCT | CGTACGTACAAAATCTTGGGATAC | 123 | AAT | 17 | 8 | 94-120 | 3 |
| VChr18a | TTCCCACCCGGTAAATATGA | CATCCAAACATCACGCTGAG | 167 | AAGG | 18 | 8 | 151-192 | 1 |
| VChr18b | ATACGCAAATGATCACAGCA | CATTTTCTCCATGGCCTCAT | 155 | AGGC | 18 | 5 | 137-154 | 1 |
| VChr18c | TGAAGCCCATTACAACCAAA | TGCAAATTAAAGCCAAGTGTG | 133 | AATC | 18 | 4 | 125-134 | 2 |
| VChr18d | TAGGTACGGTCCCAATGACC | TCGATCGATCATCTTCATCTCT | 205 | AAACT | 18 | 3 | 195-205 | 3 |
| VChr19a | TGGATTCACCATTGTCCTCA | CGAGGATACCAACAAGAATGAA | 143 | AAG | 19 | 10 | 121-150 | 1 |
| VChr19b | TTTGTTAGGTGTTGTTACCCGTTA | ATCTTCTGGCCATGTGGTTC | 170 | AGAT | 19 | 5 | 157-171 | 1 |

The marker VChr10b amplify the same locus of the marker VMC 4f-1
