# Supplementary material for: A set of microsatellite markers with long core repeat optimized for grape (Vitis spp.) genotyping
Source: BMC Plant Biol. 2008 Dec 16;8:127. doi: 10.1186/1471-2229-8-127 (PMC2625351; doi:10.1186/1471-2229-8-127)
Supplement: Additional file 4 — Summary of statistics for the 45 SSR markers developed in grape. [file 1471-2229-8-127-S4.doc]

Additional file 4: Summary of statistics for the 45 SSR markers developed in grape.

| Locus | LG | N of alleles | N of genotypes | h Obs | h Exp | PIC | NE-I | NE-SI | HW | Null alleles |
| --- | --- | --- | --- | --- | --- | --- | --- | --- | --- | --- |
| VChr1a | 1 | 9 | 48 | 0.458 | 0.545 | 0.525 | 0.227 | 0.537 | NS | 0.103 |
| VChr1b | 1 | 6 | 48 | 0.771 | 0.689 | 0.635 | 0.148 | 0.446 | NS | -0.076 |
| VChr1c | 1 | 3 | 48 | 0.521 | 0.476 | 0.369 | 0.382 | 0.610 | NS | -0.053 |
| VChr2a | 2 | 3 | 48 | 0.542 | 0.405 | 0.328 | 0.432 | 0.658 | ND | -0.154 |
| VChr2b | 2 | 6 | 48 | 0.563 | 0.508 | 0.467 | 0.283 | 0.569 | NS | -0.073 |
| VChr2c | 2 | 5 | 48 | 0.521 | 0.490 | 0.443 | 0.307 | 0.584 | ND | -0.056 |
| VChr3a | 3 | 14 | 48 | 0.688 | 0.836 | 0.810 | 0.047 | 0.348 | NS | 0.101 |
| VChr4a | 4 | 7 | 48 | 0.563 | 0.644 | 0.585 | 0.183 | 0.477 | NS | 0.074 |
| VChr5a | 5 | 11 | 48 | 0.750 | 0.772 | 0.745 | 0.074 | 0.387 | NS | 0.005 |
| VChr5b | 5 | 10 | 48 | 0.750 | 0.792 | 0.754 | 0.077 | 0.377 | NS | 0.016 |
| VChr5c | 5 | 7 | 48 | 0.729 | 0.747 | 0.704 | 0.104 | 0.406 | NS | -0.003 |
| VChr6a | 6 | 4 | 47 | 0.532 | 0.572 | 0.502 | 0.252 | 0.530 | NS | 0.018 |
| VChr7a | 7 | 3 | 48 | 0.417 | 0.506 | 0.386 | 0.364 | 0.591 | NS | 0.092 |
| VChr7b | 7 | 6 | 48 | 0.688 | 0.703 | 0.651 | 0.137 | 0.436 | NS | -0.002 |
| VChr7c | 7 | 3 | 48 | 0.688 | 0.620 | 0.532 | 0.231 | 0.501 | NS | -0.061 |
| VChr8a | 8 | 12 | 47 | 0.596 | 0.835 | 0.805 | 0.052 | 0.350 | ND | 0.167 |
| VChr8b | 8 | 16 | 48 | 0.646 | 0.889 | 0.870 | 0.025 | 0.316 | ND | 0.148 |
| VChr9a | 9 | 8 | 47 | 0.787 | 0.809 | 0.776 | 0.064 | 0.366 | NS | 0.002 |
| VChr9b | 9 | 10 | 48 | 0.313 | 0.865 | 0.840 | 0.037 | 0.331 | ND | 0.462 |
| VChr10a | 10 | 8 | 48 | 0.271 | 0.647 | 0.618 | 0.151 | 0.468 | * | 0.398 |
| VChr10b | 10 | 5 | 48 | 0.813 | 0.703 | 0.635 | 0.154 | 0.441 | NS | -0.083 |
| VChr11a | 11 | 6 | 48 | 0.542 | 0.558 | 0.506 | 0.247 | 0.535 | NS | 0.038 |
| VChr11b | 11 | 5 | 42 | 0.690 | 0.770 | 0.720 | 0.097 | 0.394 | ND | 0.054 |
| VChr12a | 12 | 7 | 48 | 0.542 | 0.695 | 0.640 | 0.146 | 0.442 | NS | 0.107 |
| VChr12b | 12 | 2 | 48 | 0.250 | 0.449 | 0.346 | 0.407 | 0.630 | NS | 0.280 |
| VChr13a | 13 | 7 | 48 | 0.625 | 0.698 | 0.652 | 0.135 | 0.438 | NS | 0.066 |
| VChr13b | 13 | 8 | 48 | 0.500 | 0.643 | 0.613 | 0.156 | 0.471 | NS | 0.141 |
| VChr13c | 13 | 5 | 48 | 0.750 | 0.744 | 0.689 | 0.117 | 0.411 | NS | -0.010 |
| VChr13d | 13 | 4 | 48 | 0.250 | 0.248 | 0.234 | 0.581 | 0.773 | ND | 0.005 |
| VChr14a | 14 | 3 | 48 | 0.500 | 0.541 | 0.444 | 0.307 | 0.559 | NS | 0.031 |
| VChr14b | 14 | 15 | 47 | 0.234 | 0.811 | 0.786 | 0.056 | 0.362 | *** | 0.561 |
| VChr15a | 15 | 8 | 46 | 0.717 | 0.728 | 0.677 | 0.121 | 0.420 | NS | -0.006 |
| VChr15b | 15 | 10 | 48 | 0.292 | 0.830 | 0.799 | 0.054 | 0.353 | ND | 0.468 |
| VChr16a | 16 | 8 | 48 | 0.604 | 0.632 | 0.602 | 0.164 | 0.478 | NS | 0.034 |
| VChr16b | 16 | 7 | 48 | 0.500 | 0.558 | 0.526 | 0.227 | 0.531 | NS | 0.081 |
| VChr16c | 16 | 4 | 47 | 0.638 | 0.582 | 0.516 | 0.240 | 0.522 | NS | -0.050 |
| VChr17a | 17 | 3 | 45 | 0.178 | 0.463 | 0.372 | 0.380 | 0.616 | ND | 0.437 |
| VChr17b | 17 | 3 | 47 | 0.021 | 0.142 | 0.134 | 0.745 | 0.866 | ND | 0.657 |
| VChr17c | 17 | 8 | 48 | 0.333 | 0.548 | 0.519 | 0.233 | 0.537 | ** | 0.275 |
| VChr18a | 18 | 8 | 48 | 0.542 | 0.705 | 0.659 | 0.130 | 0.434 | NS | 0.139 |
| VChr18b | 18 | 5 | 48 | 0.521 | 0.730 | 0.672 | 0.128 | 0.421 | ** | 0.172 |
| VChr18c | 18 | 4 | 48 | 0.208 | 0.196 | 0.187 | 0.657 | 0.817 | ND | -0.047 |
| VChr18d | 18 | 3 | 44 | 0.364 | 0.411 | 0.370 | 0.389 | 0.644 | ND | 0.096 |
| VChr19a | 19 | 10 | 48 | 0.792 | 0.784 | 0.748 | 0.078 | 0.381 | NS | -0.007 |
| VChr19b | 19 | 5 | 48 | 0.500 | 0.711 | 0.652 | 0.139 | 0.433 | NS | 0.171 |

N of genotypes = number of genotypes analyzed to calculate the statistics; hObs, hExp = observed and expected heterozigosity; PIC = polymorphism index content; NE-I, NE-SI = non-exclusion probability between two unrelated individuals and between two hypothetical full sibilings

HW = Hardy Weinberg equilibrium: NS = not significant, * = significant at the 5% level, ** = significant at the 1% level, *** = significant at the 0.1% level, ND = not done

null allele = frequency of null allele
