# Supplementary material for: A set of microsatellite markers with long core repeat optimized for grape (Vitis spp.) genotyping
Source: BMC Plant Biol. 2008 Dec 16;8:127. doi: 10.1186/1471-2229-8-127 (PMC2625351; doi:10.1186/1471-2229-8-127)
Supplement: Additional file 5 — List of the alleles found in the fingerprinting of 48 grape cultivars and rootstocks of the study. [file 1471-2229-8-127-S5.doc]

Additional file 5: List of the alleles found in the fingerprinting of 48 grape cultivars and rootstocks of the study. The numbers reported represent the number of repetition of the core motif of the locus. In columns 3 and 4 represent the alleles of the cv Chardonnay and Merlot respectively and are given for reference. The allele of the sequenced genotype PN40024 are reported in bold. See the text for details.

| Locus | Motif | Chardonnay  VCR 4 | Merlot  VCR 494 |  | | | | | | | | | | | | | | | |
| --- | --- | --- | --- | --- | --- | --- | --- | --- | --- | --- | --- | --- | --- | --- | --- | --- | --- | --- | --- |
|  |  | Alleles | | | | | | | | | | | | | | | | | |
| VChr1a | ATCC | 7/12+2 | 7/7 | N.D. | 3+1 | 5 | 6 | **7** | 8+1 | 9+1 | 11+1 | 12+2 |  |  |  |  |  |  |  |
| VChr1b | ATCC | 4/7 | 4/7 | 1+3 | 3 | 4 | 5 | 6 | **7** |  |  |  |  |  |  |  |  |  |  |
| VChr1c | AGCC | 4/6 | 4/6 | 2+3 | 4 | **6** |  |  |  |  |  |  |  |  |  |  |  |  |  |
| VChr2a | AGGC | 5/5 | 2+3/5 | 2+3 | **5** | 7+1 |  |  |  |  |  |  |  |  |  |  |  |  |  |
| VChr2b | AGCT | 3/5 | 4/5 | 3 | 4 | **5** | 6 | 7 |  |  |  |  |  |  |  |  |  |  |  |
| VChr2c | AGCC | 5+3/5+3 | 5+3/5+3 | **5** | 5+1 | 5+3 | 6+2 | 7+3 |  |  |  |  |  |  |  |  |  |  |  |
| VChr3a | AAT | 17/17 | 11/17 | 9 | 10 | 11 | 12 | 13 | 14 | 15 | 16 | **17** | 20+1 | 24+1 | 26+1 | 31+1 | 33+2 |  |  |
| VChr4a | AAAG | 11/6+1 | 14/14 | 2+2 | 3+1 | 4+1 | 6+1 | 7 | **8** | 10 |  |  |  |  |  |  |  |  |  |
| VChr5a | AGATG | 15+3/18 | 15+3/18 | 4 | 6 | **7** | 10+3 | 13+3 | 14+4 | 15+3 | 16+3 | 17+3 | 18 | 19+1 |  |  |  |  |  |
| VChr5b | AAAG | 4/7 | 4/7+3 | 3 | 4 | 4+3 | 6 | 6+2 | **7** | 7+3 | 8+1 | 11 | 13 |  |  |  |  |  |  |
| VChr5c | ACAT | 2+1/2+1 | 2+1/2+1 | N.D. | 2+1 | 3+1 | 4+1 | 6 | 7 | **8** |  |  |  |  |  |  |  |  |  |
| VChr6a | AATC | 3+1/5 | 5/6 | 3+1 | 4 | 5 | **6** |  |  |  |  |  |  |  |  |  |  |  |  |
| VChr7a | AAAAG | 5/6 | 5/6 | 3+1 | 5 | **6** |  |  |  |  |  |  |  |  |  |  |  |  |  |
| VChr7b | ACAT | 9/9 | 7/7 | 5 | 6 | 7 | 8 | **9** | 10+3 |  |  |  |  |  |  |  |  |  |  |
| VChr7c | ATGC | 4+1/6 | 6/6 | 4+1 | 5 | **6** |  |  |  |  |  |  |  |  |  |  |  |  |  |
| VChr8a | AAT | 11/22+1 | 19/22+1 | **11** | 12 | 13 | 14 | 15 | 15+1 | 17 | 17+2 | 18+2 | 19 | 19+2 | 22+1 |  |  |  |  |
| VChr8b | AAG | 20/23 | 9/11 | N.D. | 8 | 9 | 11 | 12 | 13 | 16 | 17 | 18 | 20 | 22 | **23** | 24+1 | 25+1 | 26+1 | 28+1 |
| VChr9a | AAG | 4/7 | 4/4 | 4 | 7 | 8 | 10 | 11 | **12** | 13 | 14 |  |  |  |  |  |  |  |  |
| VChr9b | AAT | 7+1/13+1 | 5+1/7+1 | 3+2 | 4+2 | 5+1 | 6+1 | 7+1 | 10+1 | 11+1 | 13+1 | 17+2 | **23** |  |  |  |  |  |  |
| VChr10a | ACT | 8/8 | 8/18 | 5 | 7 | 8 | 11 | 15 | 16 | **17** | 18 |  |  |  |  |  |  |  |  |
| VChr10b | AAC | 8/11 | 8/11 | 4+1 | 5 | 8 | 9 | **11** |  |  |  |  |  |  |  |  |  |  |  |
| VChr11a | AAAG | 7+2/7+2 | 4+2/9+3 | 2+2 | 4+2 | 5+2 | 7+2 | **8**+3 | 9+3 |  |  |  |  |  |  |  |  |  |  |
| VChr11b | AGAT | 4/6 | 4/7 | 4 | 5 | 5+2 | 6 | **7** |  |  |  |  |  |  |  |  |  |  |  |
| VChr12a | AATT | 6/7+3 | 6/7+3 | 4 | 5 | **6** | 6+3 | 7+3 | 8 | 9 |  |  |  |  |  |  |  |  |  |
| VChr12b | AATT | 6/6 | 4/6 | 4 | **6** |  |  |  |  |  |  |  |  |  |  |  |  |  |  |
| VChr13a | AAAAG | 7/7 | 5+2/7 | 3+4 | 4+4 | 5+2 | 5+4 | **7** | 8 | 9+4 |  |  |  |  |  |  |  |  |  |
| VChr13b | AAAT | 7/7 | 4+3/10 | 3+3 | 4+3 | 6 | **7** | 7+2 | 8 | 9 | 10 |  |  |  |  |  |  |  |  |
| VChr13c | AAT | 6/7 | 4/7 | 4 | 5 | 6 | 7 | **11** |  |  |  |  |  |  |  |  |  |  |  |
| VChr13d | AATC | 6/6 | 6/6 | 5 | **6** | 7 | 9+1 |  |  |  |  |  |  |  |  |  |  |  |  |
| VChr14a | AATC | 19+1/19+1 | 19+1/19+1 | 4 | **5** | 19+1 |  |  |  |  |  |  |  |  |  |  |  |  |  |
| VChr14b | ATC | 11+1/11+1 | 23+1/29+1 | 9+1 | 10 | 11 | 11+1 | 12 | 13 | **15** | 17+1 | 17+2 | 20+1 | 22+1 | 23+1 | 25+2 | 29+2 | 31+2 |  |
| VChr15a | ATCC | 1+2/7 | 7/7 | 1+2 | 3+2 | 4+3 | 5+3 | **7** | 8 | 10 | 11 |  |  |  |  |  |  |  |  |
| VChr15b | AAT | 6+2/16 | 9+2/16 | 4+1 | 6+2 | 7+1 | 9+2 | 14 | 15 | **16** | 22+1 | 23+1 | 24+2 |  |  |  |  |  |  |
| VChr16a | AAAT | 18/19+3 | 4+3/5+3 | 3 | 3+2 | 4 | 4+3 | 5+3 | 7+2 | **18** | 19+3 |  |  |  |  |  |  |  |  |
| VChr16b | AATT | 2/7+3 | 2/8 | 2 | 5 | 5+3 | 7 | 7+3 | **8** | 9 |  |  |  |  |  |  |  |  |  |
| VChr16c | AATT | 4+1/6 | 6/6 | 3+2 | 4+1 | 4+3 | **6** |  |  |  |  |  |  |  |  |  |  |  |  |
| VChr17a | AACC | 7/7 | 5/7 | 3+2 | 5 | **7** |  |  |  |  |  |  |  |  |  |  |  |  |  |
| VChr17b | ACTC | 6/6 | 6/6 | 4 | 5 | **6** |  |  |  |  |  |  |  |  |  |  |  |  |  |
| VChr17c | AAT | 10/12 | 10/12 | 3 | 5 | 6 | 7 | 9 | 10 | 11 | **12** |  |  |  |  |  |  |  |  |
| VChr18a | AAGG | 7/7 | 6/9 | 3+3 | 4+3 | 6 | 7 | **8** | 9 | 11 | 14 |  |  |  |  |  |  |  |  |
| VChr18b | AGGC | 3+3/3+3 | 3+3/3+3 | 1+2 | 3+3 | 4 | 4+3 | **6** |  |  |  |  |  |  |  |  |  |  |  |
| VChr18c | AATC | 5/5 | 5/5 | 3+3 | 4+2 | **5** | 6 |  |  |  |  |  |  |  |  |  |  |  |  |
| VChr18d | AAACT | 6/6 | 6/6 | 4 | 5 | **6** |  |  |  |  |  |  |  |  |  |  |  |  |  |
| VChr19a | AAG | 3+2/10 | 10/11 | 3+2 | 4+2 | 5+2 | 6+2 | 7+2 | 8+2 | 10 | **11** | 12 | 13+1 |  |  |  |  |  |  |
| VChr19b | AGAT | 7/7 | 6/7 | 4+2 | 5 | 6 | 7 | **8** |  |  |  |  |  |  |  |  |  |  |  |
